# 16. DEVELOPMENTAL BIOMARKERS OF ENVIRONMENTAL ADVERSITY AND EPIGENETIC RISK FOR MAJOR PSYCHIATRIC DISORDERS: CLUES TO PATHOGENESIS

**DOI:** 10.1093/schbul/sby014.059

**Published:** 2018-04-01

**Authors:** Kim Do

**Affiliations:** Center for Psychiatric Neuroscience, Lausanne University Hospital

## Abstract

**Overall Abstract:** Interaction between genetic risks and environmental adversities such as childhood trauma may underlie the etiology of psychiatric disorders. Mechanisms underpinning these interactions are poorly understood. This panel will present new data supporting a model in which epigenetic modifications and increased oxidative stress lead to altered trajectories and patients’ stratification. Helen Fisher will introduce the field, how exposure to severe stress may have immediate as well as long-lasting damaging effects on learning, behaviour, and health, and has been implicated in the development of psychosis. She will present epigenome-wide analyses of poly-victimisation across childhood and adolescence, utilising data from the Environmental Risk (E-Risk) Longitudinal Twin Study, an epidemiological study of 2,232 children (1,116 twin pairs) born in 1994–1995 in England and Wales and followed to 18 years of age. Results related to associations with DNA methylation in peripheral blood at age 18 years, specific forms of victimization and candidate genes in the stress response will be discussed. Luis Alameda will show that childhood trauma (sexual, physical and emotional abuse) engages oxidative stress in a cohort of early psychosis patients (n=118). Patients with high peripheral oxidation status were found to have smaller hippocampal volumes and more severe clinical symptoms, while those with lower oxidation status evidenced a compensatory antioxidant regulation and better cognition. Linear discrimination analysis highlights blood oxidation status together with childhood trauma as markers for poorer psychopathological profile, allowing patients’ stratification. Oussama Kebir will present the first longitudinal prospective study of genomic DNA methylation during psychotic transition in help-seeking young individuals (14 converters, 25 non converters). Alterations in gene promoters and pathways relevant for psychosis, including oxidative stress regulation, axon guidance and inflammatory pathways were observed. In particularly, antioxidant defense gene GTSTM5 is differentially methylated through time and two other genes of GST family might be differentially methylated after conversion to psychosis. These findings suggest that conversion to psychosis may depend on the specific control of oxidative metabolism and regulation between these genes. Darina Czamara will report on how cord blood methylation is affected by genetic and prenatal environment. Analysis on SNP (G) and environmental (E) effects as well as on GxE and G+E were performed using Illumina’s Human Omni Express Exom as well as the 450k DNA methylation array in a cohort of 817 Finnish newborns. G and the combination of G and E explained DNA methylation best, environment alone was almost never the best predictor. Furthermore, epigenetic gestational age was associated with prenatal environment as well as with childhood psychiatric problem at age 3 indicating that it might be used as a potential biomarker.

Overall, these new results suggest new biomarkers of environmental and genetic adversity that are related to pathogenic mechanisms, including epigenetic, oxidative stress and structural abnormalities, in epidemiological studies and psychiatric disorders. These biomarkers offer the potential for individualized early intervention and preventive strategies.

